# Vapor Pressures of Ruthenium and Osmium

**DOI:** 10.6028/jres.068A.031

**Published:** 1964-06-01

**Authors:** N. J. Carrera, R. F. Walker, E. R. Plante

## Abstract

The vapor pressures and heats of sublimation of ruthenium and osmium have been measured using a microbalance technique based on the Langmuir method. Heats of sublimation at 298 °K were calculated with the aid of free energy functions. Least squares lines for the vapor pressure data, heats of sublimation, and normal boiling points were obtained as follows :

1. Ruthenium:
  $$\text{Log}\;P_{\text{atm}}=7.500-\frac{32,769}{T}\left(1940-2377{}^{\circ}K\right)$$
  $$\begin{array}{l} \Delta H_{s}^{{}^{\circ}}\left(298\right)=156.1\pm 1.5\;\text{kcal}/\text{mole}\\ \text{bp}=4150\pm 100{}^{\circ}K \end{array}$$
2. Osmium:
  $$\text{Log}\;P_{\text{atm}}=7.484-\frac{39,880}{T}\left(2157-2592{}^{\circ}K\right)$$
  $$\begin{array}{l} \Delta H_{s}^{{}^{\circ}}\left(298\right)=189.0\pm 1.4\;\text{kcal}/\text{mole}\\ \text{bp}=5300\pm 100{}^{\circ}K \end{array}$$

The indicated uncertainties are estimates of the overall limits of error.

## 1. Introduction

This paper is the third in a series covering the vapor pressures of the platinum metals. Two previous papers [1, 2] 1 gave the vapor pressures and heats of sublimation of Pd, Pt, Ir, and Rh. Simultaneously with the current measurements on Ru and Os, similar measurements were carried out by Paule and Margrave [3] and by Panish and Reif [4]. In two instances (ref. 3 and sample 2 of the present paper) samples cut from the same stock were utilized in the course of the measurements on ruthenium.

Until this recent work no measurements of the vapor pressures of Ru and Os had been reported. Estimates of their heats of sublimation at 298 °K, $\Delta H_{s}^{{}^{\circ}}\left(298\right)$, were given by Brewer [5] and by Stull and Sinke [6]. The estimated and experimental values are listed in table 1.

The present results were obtained primarily by the Langmuir method, using variations of a microbalance technique previously described [1, 7, 8] and a sample heated directly by induction. In applying the Langmuir method it was assumed that the vaporization coefficients of Ru and Os are unity and that they vaporize predominantly to monatomic gaseous species over the temperature range of the measurements. Paule and Margrave [3] also used the Langmuir method, making similar assumptions, but their sample of Ru was suspended from a microbalance into a tube furnace of graphite lined with tantalum. Panish and Reif [4] made measurements by both the Langmuir and Knudsen methods which tended to confirm the assumptions and used a time-of-flight mass spectrometer in an unsuccessful search for polyatomic species. A direction-focusing mass spectrometer with a Knudsen cell was also used for measurements on Ru in the present work. Monatomic species alone were observed, and the data did not suggest that the vaporization coefficient was significantly different from unity. However, owing to the larger temperature uncertainties and the imprecision of converting observed ion currents to vapor pressures, the data are considered less accurate than the Langmuir measurements and are not reported in detail.

## 2. Experimental Technique

In principle, the equilibrium vapor pressure (*P*) of each metal at temperature *T* can be determined from its rate of sublimation in a vacuum, in accordance with the equation:

$$P=\frac{m}{\alpha }\sqrt{\frac{2\pi RT}{M}}$$

In the present work the rate of sublimation (*m*, g cm^−2^ sec^−1^) was measured by suspending a sample of known surface area from the microbalance. The vaporization coefficient (*α*) was taken to be unity, and the molecular weight of the vapor species (*M*) is that of the monomer. The value of the gas constant *R* used in the calculation of $\Delta H_{s}^{{}^{\circ}}$ is 1.98726 cal/deg mole. Actual vapor pressures would be somewhat lower than calculated if polymeric species were involved.

### 2.1. Apparatus

Two pieces of apparatus were used for the measurements. The first of these, which was used only for the first series of measurements on ruthenium, was the oil-pumped vacuum microbalance apparatus described previously [1,5]. A significant change was, however, made in the method of temperature measurement, as discussed in section 2.4 below.

In general principle the second apparatus, which was used for the remaining four series of measurements on ruthenium and for the series on osmium, was similar to the first apparatus, with the exception that it was ion pumped. This second apparatus has also been described in some detail recently [8], and only the essential features will be repeated here.

The sample was suspended from an equi-arm, quartz beam microbalance by a chain of 0.025 cm diam sapphire rods. An 0.025 cm diam hole through one end of the sample allowed a short loop of fine wire (0.005 cm diam iridium for the Ru sample and 0.0125 cm diam tungsten for the Os sample) to pass through the sample and hang over the hook of the lowest sapphire suspension rod. The presence of the hole and the loop (which was not heated significantly by the induction field) was ignored when calculating the effective surface area of the sample.

The sample hung inside a glass tube, about 13 mm in diam; a water-cooled radiofrequency coil fitted closely around the tube and coupled directly with the sample. A fused quartz sleeve was fitted inside the glass tube to collect the sublimed metal.

The apparatus was pumped continuously throughout each run with an ion pump rated by the manufacturer at 90 liter/sec. Pressures were measured at the pump by the current drawn by the pump.

For the first three series of runs on ruthenium a 450 kc/s, 50 kw radio frequency generator was used to heat the samples. The long term stability of the output of the generator was not adequate for runs lasting several hours. Hence, a more stable 2 to 3 Mc/s, 2 kw generator was employed for the last two series on ruthenium and the series on osmium, during which more extended runs were made.

### 2.2. Samples

Two ruthenium samples having different impurity contents were used in the measurements. The first and purest sample, referred to as sample 1, was used for one series of measurements on each of the two microbalance systems mentioned above. It consisted of a short rod about 1.9 cm long by 0.32 cm diam. The sample was prepared by swaging and heating in vacuo to 1450 °C. At the conclusion of the series 1 experiments on sample 1, remeasurement of the sample dimensions showed that some volume expansion of the sample had occurred. This was presumably due to recrystallization of the sample. As a result, the porosity increased during the experiments from an initial value close to zero to a final value of about 10 percent while the area increased by about 13 percent. The average of the initial and final areas was used as the room temperature area of the sample.

The purity of sample 1 was determined by spectrochemical analysis before each series of runs. A summary of the results of the analysis is shown in table 2. The International Nickel Co., which donated the sample, furnished the analysis of the sample as used at the start of the first series of measurements. After this series the sample was analyzed again at NBS. It will be noted in table 2 that some purification of the sample occurred during the series, but the absence of Os in the second analysis is unexplained. Other small discrepancies in the impurity contents are probably attributable to the limited precision of the spectrochemical technique.

The second ruthenium sample (referred to as sample 2) was initially used as a rectangular bar, cur from the same stock as was used by Paule and Margrave [3]. The stock appeared to have been prepared by cold pressing and sintering and retained some porosity. As used for the third and fourth series of measurements on Ru, the bar was about 1.14 cm long with about a 0.39 cm square cross section. For the fifth series of measurements the bar was remachined to a rod of the same length with a diameter of about 0.37 cm. Table 2 gives an analysis of the stock material as received.

The osmium sample was 0.23 cm diam and 1.47 cm long; it was machined from a larger, cold-pressed and sintered bar. As received, the material was not particularly pure, but was purified by heating to a high temperature (~2000 °C) in a vacuum for an extended period to vaporize off the more volatile impurities. The analysis shown in table 2 was made on the sample after purification and before the series of measurements of its rate of vaporization. It may be presumed that further puricfiation occurred during the series, but no effects attributable to this cause were detected.

Samples were hung in the coil with their principal axes vertical. The surface areas at the run temperatures were calculated from their overall geometry, using literature values for the thermal expansion coefficients. No corrections were applied for the slight change in surface area due to sublimation. Assuming each sample sublimed uniformly over its surface area, the error introduced by neglecting this change in the surface area was less than 0.3 percent.

### 2.3. Procedure

The experimental procedure was generally similar to that used previously. The micro balance was used as a deflection instrument, deflections of its beam being directly proportional to changes in mass of the sample. Beam deflections were measured with a cathetometer readable to 1 *μ* displacements. The microbalance was calibrated, with each sample *in situ*, using Class M microbalance weights previously calibrated by the Mass Section of NBS.

Interaction between the rf field and the sample caused the balance to be displaced full scale. Therefore, the balance was first read with the sample at room temperature. The sample was then heated rapidly, held at constant temperature for a given length of time, then cooled rapidly. When the sample had returned to room temperature, the balance was then read again to obtain the total mass change during the course of each run. As explained previously [1], no significant weight loss occurred during the short heating and cooling periods.

The duration of the runs ranged from ½ min at the highest temperature to over 7 hr at the lowest for ruthenium, and 3 min to 52 hr for osmium. The uncertainty in the measurement of the weight losses was about ±4 percent for a weight loss as small as 25 *μ*g, but was proportionately smaller for larger weight losses.

For the oil-pumped apparatus pressures were in the range 2×10^−6^ to 8×10^−5^ torr throughout each run, as discussed previously [1]. The ion-pumped apparatus permitted pressures of 1×10^−9^ torr to be obtained initially in the cold system. During the first heating of a series, the pressure generally rose to a maximum of about 5×10^−8^ torr. Subsequent runs yielded successively lower maximum pressures, so that during the later runs of a series the pressure was maintained continuously within the range 1 to 5×10^−9^ torr. No trends in the data with changes in pressure were detected.

### 2.4. Temperature Measurement

A common source of error in measurements by the Langmuir method arises from uncertainties in the emittance of the surface of the sample, and hence, in the conversion of observed brightness temperatures to thermodynamic temperatures. A method of introducing a blackbody hole into the sample was described previously [2] for the measurements on palladium. For a substance having a vaporization coefficient of unity the presence of the hole does not increase the effective surface area of the sample and eliminates the emittance problem. In the present measurements an easier-to-use method of introducing the hole was employed.

A hole having a length: diameter ratio of 10 or greater was drilled into the bottom of each sample along its principal axis. The diameters of the holes were in the range 0.076–0.102 cm, depending on the lengths permitted by the overall size of the samples. An NBS calibrated pyrometer with a magnifying objective was used to measure temperatures by sighting up the holes through a calibrated window (with magnetic shutter) and with the aid of a calibrated mirror. The window and mirror calibrations were checked periodically.

Using this technique, the principal source of error in the temperature measurements arose from the instability of the rf generators, causing temperature fluctuations. The fluctuations were normally within the range ±5 °C of the temperatures given in the tables of results.

One additional small, but undetermined source of temperature error was inadvertently introduced for the second series of measurements on sample 1 of Ru. After the first series, a piece was broken from the bottom of the sample for analysis. In lengthening the blackbody hole, the drill broke through the 0.025 cm hole, close to the top of the sample, which was used for suspension purposes. Observations confirmed that the hole did not meet blackbody conditions, but the extent of the departure could not be determined with precision.

## 3. Results and Discussion

### 3.1. Ruthenium

The results of the five series of measurements on the two samples of ruthenium are given in table 3. Calculated values of the vapor pressures and the third law heats of sublimation are also given in the table. Adjusted values of the free energy functions given by Stull and Sinke [6] were used to obtain the heats. Clusius and Piesbergen [9] give for the normal entropy of solid ruthenium at 298 °K: $S_{298}^{0}=6.82\pm 0.05$ cal deg^−1^ mole^−1^. This value was used to adjust the values of $S_{T}^{0}$ and $-\left(F_{T}^{0}-H_{298}^{0}\right)/T$ listed by Stull and Sinke by −0.08 cal deg^−1^ mole^−1^. Table 4 summarizes the data of table 3 in the form of the mean third law heats and the standard deviations for each series of runs. A second law heat of sublimation and standard deviation was computed from the least squares line through a plot of ln *P* versus 1/*T* for each series of runs. The second law values were referred to 298 °K using the $H_{T}^{0}-H_{298}^{0}$ values of Stull and Sinke and are given in table 4. Also shown in this table are the second and third law heats of sublimation obtained by treating all the data of the five series as a unit.

**Figure 1:**
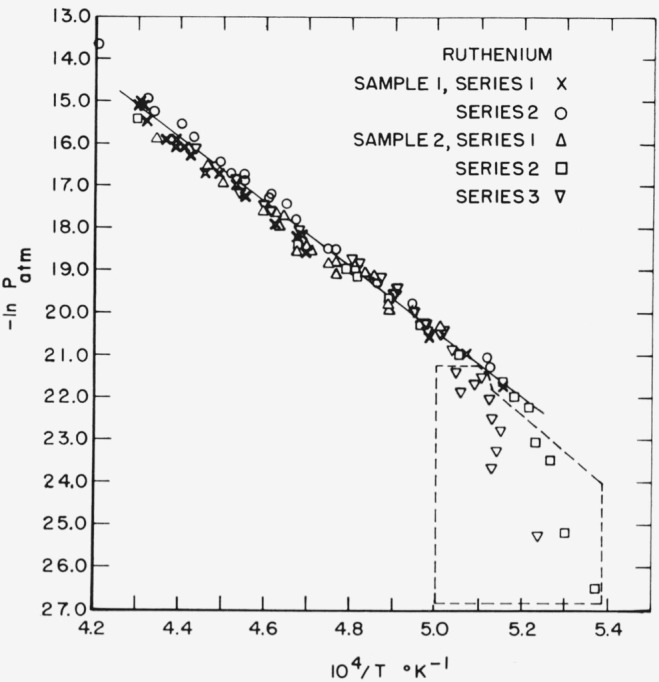
Logarithm of calculated vapor pressures of ruthenium versus reciprocal of absolute temperature. All data enclosed by the broken line were rejected.

Figure 1 shows a plot of —ln *P* versus 1/T for the five series of runs. The least squares line representing all the data for the temperature range 1940 to 2377 °K is given by:

$$\text{Log}\;P_{\text{atm}}=7.500-\frac{32,769}{T}.$$

A normal boiling point of 4150± 100 °K is obtained by using the third law heat of sublimation and extrapolating the free energy function data of Stull and Sinke [6].

As indicated in table 3, a total of 14 runs during the last two series on sample 2 were rejected in calculating the results given in table 4. The reasons for the rejection may be briefly summarized as follows.

With a view to obtaining second law heats of vaporization that were closer to the third law values, an attempt was made to extend the temperature range over which the measurements were made. In particular, attempts were made during both series to extend the temperature range downward. As is apparent from figure 1, however, it was found that below a vaguely defined temperature the rates of vaporization were unexpectedly low, and the calculated vapor pressures departed markedly from the straight line ln *P* versus 1/*T* plot of the data obtained at higher temperatures. There was some indication that the temperature at which this departure was detected increased with more extended heating of the sample. Thus, no cause was found to reject the data of the first run of sample 2, series 2 which were obtained at 1942 °K. Only data of this series which were obtained at 1912 °K or below were rejected. During series 3, however, it appeared necessary to reject all data obtained at 1981 °K and below. (In order to be consistent, one later value obtained at 1959 °K was rejected, even though the data yielded a heat of sublimation that was within the range of the accepted values.)

The cause of the departure is at present unexplained, but the effect is very similar to an effect which was observed during measurements on platinum [1]. One possible explanation which was suggested at that time was that contamination of the surface of the sample occurred, either from its surroundings or by migration of impurities from within. In the present case, however, the surface of the sample was remachined between series 2 and 3. While impurities such as carbon or oxygen (which are not detected by the spectrochemical analyses) may contribute to the problem, further work will be necessary before its nature can be more clearly elucidated.

The relatively large uncertainty in the room temperature area, *S*o, for the sample 1, series 1 measurements can introduce a maximum uncertainty of ±0.3 kcal in individual third law heats for this series. Since this error is well within the precision of the measurements and will tend to cancel in obtaining the average third law heat, the data from this series were accepted.

The question also arises as to whether all the data of the second series of runs on sample 1 should be rejected, in view of the larger temperature error of the series which was discussed in section 2.4. As shown in table 4 this series yielded the lowest mean third law heat of sublimation, but the mean is not in disagreement with the means of the other four series, within the limits of precision. All the data of the series were, therefore, retained, and were used along with the other accepted data, to arrive at a final estimate of the heat of sublimation based on the combined data of the five series and the third law method.

The overall limits of error of the absolute value of $\Delta H_{s}^{{}^{\circ}}\left(298\right)$ were estimated by taking into account the scatter of the data, the uncertainty in the temperature (±10 °K), and the uncertainty of the weight loss measurements. No allowance was made for the uncertainty in the free energy functions or in the vaporization coefficient. The overall limits of error of the absolute value were estimated to be ±1.5 kcal/mole, and the corresponding overall limits of error of the vapor pressures are about ±35 percent. Within the limits of the uncertainty of the temperature measurement the second law value of $\Delta H_{s}^{{}^{\circ}}\left(298\right)$ for all 94 measurements (shown in table 4) is in agreement with the third law value.

The final estimate of the absolute value of $\Delta H_{s}^{{}^{\circ}}\left(298\right)$ for ruthenium is, therefore, 156.1 ±1.5 kcal/mole. The value is in agreement with the values of Paule and Margrave [3] and Panish and Reif [4] within the limits of experimental error.

### 3.2. Osmium

The vapor pressures of osmium calculated from the measured rates of vaporization are given in table 5, together with the corresponding values of $\Delta H_{s}^{{}^{\circ}}\left(298\right)$. The free energy functions of Stull and Sinke [6] were used to obtain the heats of sublimation.

**Figure 2:**
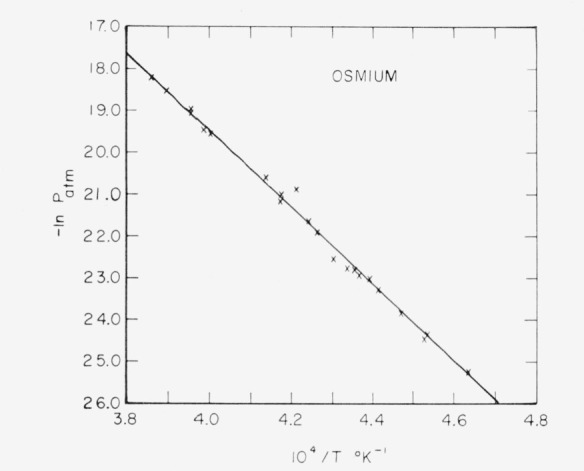
Logarithm of vapor pressure of osmium versus reciprocal of absolute temperature.

Figure 2 shows a Clausius-Clapeyron plot of the data shown in table 5. The least squares line through the data may be represented by:

$$\text{Log}\;P_{\text{atm}}=7.484-\frac{39,880}{T}$$

for the temperature range 2157 to 2592 °K. This equation leads to a second law value of $\Delta H_{s}^{{}^{\circ}}\left(298\right)$ of 184.1 kcal/mole with a standard deviation of ±3.0 kcal/mole. A normal boiling point of 5300 ± 100 °K is obtained by using the third law heat and extrapolating the free energy function data of Stull and Sinke [6].

Neglecting uncertainties in the free energy functions and assuming a vaporization coefficient of unity, the overall limits of error in the absolute value of $\Delta H_{s}^{{}^{\circ}}\left(298\right)$ were estimated to be ±1.4 kcal/mole. These limits were applied to the mean third law value to obtain a corresponding error of ±30 percent in the vapor pressures. The overall error in the temperature measurement is sufficient to account for the discrepancy between the second and third law values.

Based on the mean third law value, the best estimate of the absolute value of $\Delta H_{s}^{{}^{\circ}}\left(298\right)$ is, therefore, 189.0 ±1.4 kcal/mole. This value agrees well with the value of Panish and Reif [4] within the limits of experimental error.

## Figures and Tables

**Table 1:** Heats of sublimation of ruthenium and osmium

Substance	2d Law	$\Delta H_{s}^{{}^{\circ}}\left(298\right)$	3d Law	Reference
				
		*kcal*/*mole*		
Ruthenium		160		Brewer [5],
		144		Stull, Sinke [6].
	155.5±5		151.5±4	Paule, Margrave [3].
	151 ±10		154.9±1.3	Panish, Reif [4].
	151.6±2.1		156.1±1.5	This work.
Osmium		174		Brewer [5].
		160		Stull, Sinke [6].
	192±10		187.4±0.9	Panish, Reif [4].
	184.1±3.0		189.0±1.4	This work.

**Table 2:** Spectrochemical analyses of samples

Impurity element detected	Rutheniuir			Osmium
	Sample 1		Sample 2	
				
	INCO %	NBS %	NBS %	NBS %
Ag	……	?	?	0.0001–0.001
Al	……	?	0.0001–0.001	.0001–.001
Au	……	0	0	.0001–.001
Ba	……	—	—	.001–.01
Be	……	0	0	<0.0001
Ca	……	?	.01–.1	.01 –.1
Co	……	0	0	.001 –.01
Cr	……	0	0	.001 –.01
Cu	……	0.0001–0.001	.0001–.001	.001 –.01
Fe	0.0025	.0001–.001	.001 –.01	.01 –.1
Mg	……	<0.0001	.0001–.001	.01 –.1
Mn	……	……	0	.001 –.01
Ni	.0002	……	0	.001 –.01
Os	.02	0	0	……
Pb	.0005	?	<0.0001	.0001 –.001
Pd	<.001	?	.0001–0.001	.0001–.001
Pt	……	?	.01 –0.1	.001 –.01
Rh	.007	?	?	……
Ru	……	……	……	.001 –.01
Sb	<.001	……	0	……
Si	……	0.0001–0.001	.0001–0.001	.01 –.1
Sn	.003	0	0	.0001–.001
Estimated purity maximum	99.97%	99.997–99.9997%	99.8–99.98%	99.5–99.95%

0=Not detected. ?=Detection uncertain.

**Table 3:** Vapor pressures and heats of sublimation of ruthenium**

Temperature	Duration of run	Weight loss	Vapor pressure	$\Delta H_{s}^{{}^{\circ}}\left(298\right)$
				
Sample 1, series 1 *S*o=2.108 cm^2^				

*°K*	*min*	*μ_g_*	*atm*	*kcal/mole*
1974	30	30.3	7.72·10^−10^	155.8
2007	30	45.1	1.16·10^−9^	156.8
2081	15	111.3	5.81·10^−9^	155.9
2141	10	153.8	1.22·10^−8^	157.1
2131	7.5	77.4	8.17·10^−9^	158.1
2135	10	150.8	1.20·10^−8^	156.8
2164	8	163.1	1.63·10^−8^	157.6
2228	5	341.0	5.51·10^−8^	156.8
2317	1	333.3	2.74·10^−7^	155.6
2208	1	52.3	4.21·10^−8^	156.6
2246	1	66.2	5.37·10^−8^	158.2
2324	(40 sec)	219.5	2.71·10^−7^	156.1
2323	(25 sec)	148.2	2.93·10^−7^	155.7
2279	1	146.1	1.19·10^−7^	156.9
2198	4	156.4	3.14·10^−8^	157.2
1940	90	42.0	3.54·10^−10^	156.2
2043	15	50.8	2.63·10^−9^	156.2
2280	1	122.6	1.00·10^−7^	157.7
2275	1	126.7	1.04·10^−7^	157.2
2316	1	220.0	1.81·10^−7^	157.4
2292	2	292.3	1.20·10^−7^	157.7
2262	1.5	153.3	8.32·10^−8^	157.3

Sample 1, series 2 *S*o=1.810 cm^2^				

1954	45	38.0	7.48·10^−10^	154.4
2023	17	50.5	2.68·10^−9^	154.7
2228	2	157.4	7.41·10^−8^	155.5
2058	6	32.2	4.87·10^−9^	154.9
2214	1.5	93.0	5.82·10^−8^	155.6
2076	5	34.4	6.27·10^−9^	155.2
2308	1.5	387.2	2.47·10^−7^	155.5
2199	1.5	92.1	5.75·10^−8^	154.6
2168	2	75.1	3.49·10^−8^	154.6
2107	2.5	26.4	9.69·10^−9^	155.6
2152	2	60.4	2.80·10^−8^	154.4
2274	1.5	289.3	1.83·10^−7^	154.6
2102	3	31.7	9.69·10^−9^	155.3
2142	3	62.6	1.93·10^−8^	155.3
2171	2	70.6	3.28·10^−8^	155.1
2198	2	104.2	4.88·10^−8^	155.3
2257	1	144.0	1.36·10^−7^	154.8
2314	0.5	173.5	3.33·10^−7^	154.5
2377	0.5	592.4	1.15·10^−6^	152.8
1952	60	39.8	5.88·10^−10^	155.2

Sample 2, series 1 So=2.142 cm^2^				

2069	15	105.8	5.43·10^−9^	155.2
2060	5	31.7	4.87·10^−9^	155.0
2130	3	41.4	1.08·10^−8^	156.9
2155	2	50.6	1.98·10^−8^	156.1
2163	4	118.6	2.33·10^−8^	156.0
2242	1.5	129.3	6.88·10^−8^	156.8
2140	2	24.0	9.38·10^−9^	158.2
2098	5	34.2	5.30·10^−9^	157.5
2160	2	43.4	1.70·10^−8^	157.1
1996	30	55.2	1.39·10^−9^	155.2
2047	8	27.6	2.64·10^−9^	156.5
2098	2.5	16.9	5.23·10^−9^	157.6
2099	7	64.9	7.18·10^−9^	156.3
2048	10	33.2	2.54·10^−9^	156.8
2107	6	54.7	7.07·10^−9^	157.0
2129	2.5	29.6	9.23·10^−9^	157.5
2159	1.5	35.8	1.87·10^−8^	156.6
2222	1	60.3	4.80·10^−8^	157.0
2302	0.5	81.8	1.32·10^−7^	157.9
2178	1.5	46.5	2.44·10^−8^	156.9

Sample 2, series 2 *S*o =2.142 cm^2^				

*°K*	*min*	*μ_g_*	*atm*	*kcal/mole*
1942	50	27.4	4.09·10^−10^	155.8
1976	30	33.0	8.28·10^−10^	155.7
2017	15	31.4	1.59 10^−9^	156.3
2048	10	37.0	2.83·10^−9^	156.3
2080	5	37.0	5.71·10^−9^	155.9
2089	5	40.0	6.18·10^−9^	156.2
2139	2	26.9	1.05·10^−8^	157.6
2163	2	48.2	1.89·10^−8^	156.9
2159	2	49.2	1.93·10^−8^	156.5
2079	5	33.5	5.17·10^−9^	156.2
1885	1446	20.3	1.03·10^−11^	*165.1
1912	1000	131.8	9.78·10^−11^	*158.8
1932	135	52.7	2.91·10^−10^	156.3
1918	150	46.1	2.28·10^−10^	156.1
2328	3.25	825.4	2.06·10^−7^	157.7
1900	240	21.3	6.56·10^−11^	*159.4
1862	7295	30.4	3.05·10^−12^	*167.6

Sample 2, series 3 *S*_o_=1.568 cm^2^				

1995	20	28.2	1.46·10^−9^	155.0
2037	12	44.5	3.87·10^−9^	154.2
2075	5	33.2	6.99·10^−9^	154.7
1978	30	9.5	3.26·10^−10^	*159.5
2010	18	29.1	1.68·10^−9^	155.6
1986	60	53.6	9.21·10^−10^	156.1
1945	75	5.9	8.03·10^−11^	*162.3
1948	130	21.8	1.71·10^−10^	*159.6
2022	12	25.4	2.20·10^−9^	155.4
2054	6	28.6	4.99·10^−9^	154.5
2083	5	36.4	7.67·10^−9^	154.9
2075	6	31.4	5.51·10^−9^	155.6
2040	9	29.5	3.42·10^−9^	155.0
2008	18	24.5	1.41·10^−9^	156.1
1996	30	40.9	1.41·10^−9^	155.2
1981	30	15.0	5.15·10^−10^	*158.0
1965	90	33.6	3.83·10^−10^	*157.9
1949	180	9.5	5.40·10^−11^	*164.2
1952	120	32.7	2.79·10^−10^	*158.1
1959	110	49.5	4.61·10^−10^	*156.7
1942	314	39.5	1.28·10^−10^	*160.3
1910	430	4.5	1.06·10^−11^	*167.1
2138	3	42.7	1.52·10^−8^	156.0
2168	2.5	53.6	2.30·10^−8^	156.4
2178	2.25	56.4	2.70·10^−8^	156.4
2205	2.17	77.7	3.88·10^−8^	156.8
2208	2	83.6	4.53·10^−8^	156.3
2230	2.08	130.4	6.82·10^−8^	156.0
2255	1	102.7	1.12·10^−7^	155.5

** Experimental sequence.

* Data rejected.

**Table 4:** Heat of sublimation of ruthenium

Determination	$\Delta H_{s}^{{}^{\circ}}\left(298\right)$ and standard deviation	
	3d Law*	2d Law
		
Sample 1: 99.9997–99.997% Ru		
1. Oil pumped. 5×10^−6^ torr ○ 450 kc/s generator.	156.9±0.8	*Kcal/mole* 153.0±2.9
2. Ion pumped. 5×10^−9^ torr ○ 450 kc/s generator. Small departure from blackbody hole in sample	154.9±0.6	157.6±2.6
Sample 2: 99.98–99.8% Ru		
1. Ion pumped. □ 450 kc/s generator	156.7±0.9	143.4±5.3
2. Ion pumped. □ 2 Mc/s generator	156.4±0.5	148.3±2.2
3. Ion pumped. ○ 2 Mc/s generator	155.6±0.7	147.1±3.7
All 94 measurements	156.1 ±1.1	151.6±2.1

* Means of accepted measurements.

○Vindicates circular cross-section sample.

□indicates square cross-section sample.

The 2 Mc/s generator had a better long term stability than the 450 kc/s generator and could be used for measurements at lower temperatures.

**Table 5:** Vapor pressures and heats of sublimation of osmium*

Temperature	Duration of run	Weight loss	Vapor pressure	$\Delta H_{s}^{{}^{\circ}}\left(298\right)$
				
*°K*	*min*	*μg*	*atm*	*kcal*/*mole*
2374	60	45.4	8.58·10^−10^	186.3
2419	20	18.7	1.07·10^−9^	188.8
2528	5	23.2	5.41·10^−9^	189.0
2569	5	37.4	8.80·10^−9^	189.6
2592	3	31.6	1.24·10^−8^	189.5
2397	30	16.5	6.25·10^−10^	189.6
2396	100	63.2	7.20·10^−10^	188.9
2358	205	71.3	3.93·10^−10^	188.7
2346	105	28 1	3.01·10^−10^	189.0
2323	172	25.4	1.66·10^−10^	190.0
2305	302	36.5	1.35·10^−10^	189.4
2290	465	45.9	1.10·10^−10^	189.1
2530	5	24.0	5.62·10^−9^	189.0
2510	6	18.3	3.54·10^−9^	189.8
2500	7	19.2	3.18·10^−9^	189.6
2210	1010	22.1	2.24·10^−11^	189.3
2296	310	34.5	1.25·10^−10^	189.0
2278	303	28.3	1.05·10^−10^	188.4
2267	543	38.5	7.91·10^−11^	188.7
2237	732	30.1	4.56·10^−11^	188.7
2205	1390	32.7	2.60·10^−11^	188.5
2157	3136	32.3	1.12·10^−11^	188.0

Mean and standard deviation				189.0±0.8

* Experimental sequence.

*S*o =1.141 cm^2^.

## Footnotes

1 Figures in brackets indicate the literature references at the end of this paper

## References

[1] Hampson RF, Walker RF (1961). J Res NBS.

[2] Hampson RF, Walker RF (1962). J Res NBS.

[3] Paule RC, Margrave JL (1963). J Phys Chem.

[4] Panish MB, Reif L (1962). J Chem Phys.

[5] Brewer L, Quill LL (1950). The chemistry and metallurgy of miscellaneous materials: thermodynamics.

[6] Stull DR, Sinke GC (1956). Thermodynamic properties of the elements. Am Chem Soc.

[7] Walker RF, Katz MJ (1961). Vacuum microbalance techniques.

[8] Carrera NJ, Walker RF, Nalley W, Steggerda C, Behrndt K (1963). Vacuum microbalance techniques.

[9] Clusius K, Piesbergen U (1959). Z Naturforsch.

